# Mechanochromic Reconfigurable Metasurfaces

**DOI:** 10.1002/advs.201900974

**Published:** 2019-09-12

**Authors:** Artemios Karvounis, Nikolaos Aspiotis, Ioannis Zeimpekis, Jun‐Yu Ou, Chung‐Che Huang, Daniel Hewak, Nikolay I. Zheludev

**Affiliations:** ^1^ Optoelectronics Research Centre University of Southampton Southampton SO17 1BJ UK; ^2^ Optical Nanomaterial Group Institute for Quantum Electronics ETH Zurich 8093 Zurich Switzerland; ^3^ Centre for Disruptive Photonic Technologies & The Photonics Institute School of Physical and Mathematical Sciences Nanyang Technological University Singapore 637371 Singapore

**Keywords:** mechanochromism, metasurfaces, nanomechanics, photonic metamaterials, van der Waals materials

## Abstract

The change of optical properties that some usually natural compounds or polymeric materials show upon the application of external stress is named mechanochromism. Herein, an artificial nanomechanical metasurface formed by a subwavelength nanowire array made of molybdenum disulfide, molybdenum oxide, and silicon nitride changes color upon mechanical deformation. The aforementioned deformation induces reversible changes in the optical transmission (relative transmission change of 197% at 654 nm), thus demonstrating a giant mechanochromic effect. Moreover, these types of metasurfaces can exist in two nonvolatile states presenting a difference in optical transmission of 45% at 678 nm, when they are forced to bend rapidly. The wide optical tunability that photonic nanomechanical metasurfaces, such as the one presented here, possess by design, can provide a valuable platform for mechanochromic and bistable responses across the visible and near infrared regime and form a new family of smart materials with applications in reconfigurable, multifunctional photonic filters, switches, and stress sensors.

## Graphical abstract

**Mechanochromic response** is presented as a method to tune optical properties of photonic metasurfaces. A subwavelength array of silicon nitride/molybdenum oxide and molybdenum disulfide nanowires due to heat‐activated mechanical strain alters its optical properties drastically in the visible part of the spectrum with transmission change up to 197% and mechanically bistable response.

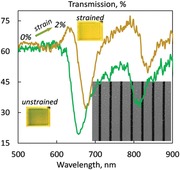

Up to date, control of electromagnetic properties of photonic metamaterials and/or metasurfaces, artificial media structured on the subwavelength scale, have been achieved via nanomechanical reconfiguration of its building blocks,^1^, ^2^, ^3^ structural phase change in the material of constituting elements,^4^, ^5^, ^6^, ^7^ carrier injection effects,^8^, ^9^, ^10^, ^11^ application of liquid crystals,^12^, ^13^ chemical modification,^14^ and by stretching the elastic substrate supporting plasmonic or dielectric metamolecules.^15^, ^16^

Mechanochromism is the color change, upon mechanically induced reorganization of crystal structure or mechanically induced structural phase transition.^17^ Such effects have been studied in a number of materials,^17^, ^18^ where strongest effects are seen in polymers,^19^ liquid crystal elastomers,^20^, ^21^, ^22^ nanofibers,^23^ and photonic crystals.^24^ Recently, chromic effects have been used as tuning mechanisms for photonic metasurfaces and plasmonics related devices with thermochromic and electrochromic responses, respectively.^25^, ^26^

In recent years, strain engineering of optical and mechanical properties of solids, in particular silicon and 2D solids have attracted considerable attention.^27^, ^28^, ^29^, ^30^, ^31^, ^32^, ^33^ 2D materials, such as graphene, oxides, nitrides, and transition metal dichalcogenides are of particular interest as constitutive elements for reconfigurable metamaterials and metasurfaces, as the extreme electron confinement inherits them with unique dielectric properties that can be controlled by external stimuli.^32^, ^34^

In particular, molybdenum disulfide, MoS_2_ is formed from the covalent bond between transition metal atoms (Mo) sandwiched by two layers of chalcogen atoms (sulfur), while every sheet is bound via weak van der Waals interaction. Several theoretical reports have indicated that the energy bandgap renormalization can occur on MoS_2_ upon stress, where microscopic parameters like carrier mobility and effective mass of carriers upon mechanical deformation can lead to substantial changes on refractive index. Furthermore, excitonic peak emission wavelengths have been observed to be sensitive to mechanical stress in its monolayer form.^35^, ^36^, ^37^, ^38^, ^39^, ^40^ MoS_2_ has excellent mechanical properties because its large Young modulus (330 GPa) and high elastic limit which makes it an attractive material for nanomechanical and mechanochromic devices.^41^

In this paper, we introduce a previously unexplored mechanism of tuning the optical properties of photonic metasurfaces that exploits the phenomenon of mechanochromism. We show that elastic strain in a MoS_2_/MoO_3−_ *_x_*/Si_3_N_4_ nanomechanical photonic metasurface causes a profound change in its optical properties, which originates from the strain‐sensitive refractive index of MoS_2_, enhanced by electromagnetic resonances created by nanostructuring. Specifically, heat activated nanomechanical deformation of the array of nanowires lead to profound reversible changes of its transmission, reflection and absorption in the visible part of the spectrum operating either as a photonic filter or as a mechanically bistable element. Transmission changes up to 197% are obtained at 654 nm upon 2% of mechanical strain, while two nonvolatile states presenting a difference in optical transmission of 45% achieved at 675 nm. The response of the system is controlled via the speed of induced mechanical stress. This type of devices can serve not only as photonic elements but also as strain sensors with an optical readout. Recently, more trilayer metasurfaces have been reported.^42^

The metasurface was fabricated on a 90 nm thick Si_3_N_4_ membrane which was patterned as an array of nanowires 22 µm long, 400 nm wide with gaps of 100 nm separating them (see **Figure** **1**). The prepatterned Si_3_N_4_ membrane, was coated with a 60 nm thick layer of MoO_3−_ *_x_* and a 5 nm layer of MoS_2_ by atmospheric pressure chemical vapor deposition (APCVD). Figure 1a shows a top view of the metasurface, while the insets present a cross‐section of the trilayer film. The white dots seen on the scanning electron microscopy (SEM) are caused by the film growth and device fabrication process; The short term roughness of the film is in the order of 1 MoS_2_ layer whereas the suspected contamination adds a waviness in the order of 3 nm (rms), verified by atomic force microscopic (AFM) measurements. The period of the contamination is in the order of 2–4 µm so this is beyond our wavelength of interest. The height of the individual contaminants is a maximum of 13 nm so with the refractive indices of our materials this translates to less than 1/10 of the wavelength of interest so it can be considered optically flat. More details related with transmission electron microscopy (TEM) and X‐ray power diffraction (XRD) analysis of MoS_2_ films grown via APCVD method can be found in our previous publication,^43^ while the growth methods of the films are included in the Experimental Section. The composition of the sample has been evaluated using reflective Raman spectroscopy. Figure 1b shows the Raman spectrum of the nanowire indicating its crystallinity: the 381 cm^−1^ line relates to the in‐plane vibration mode, while the 407.5 cm^−1^ line corresponds to the out‐of‐plane vibration A_1g_ mode of MoS_2_.^44^ The 353 and 733 cm^−1^ lines indicate the presence of MoO_3−_ *_x_* in the structure.^45^ Furthermore, we measured the complex refractive index of MoS_2_ layer of the composite nanomembrane by spectral ellipsometry (Figure 1c). The refractive index of our films is similar to previous literature,^46^ demonstrating MoS_2_ as a high index dielectric material. These results were used to computationally model the optical properties of the nanowire array, as presented in **Figure** **2**b.

**Figure 1:**
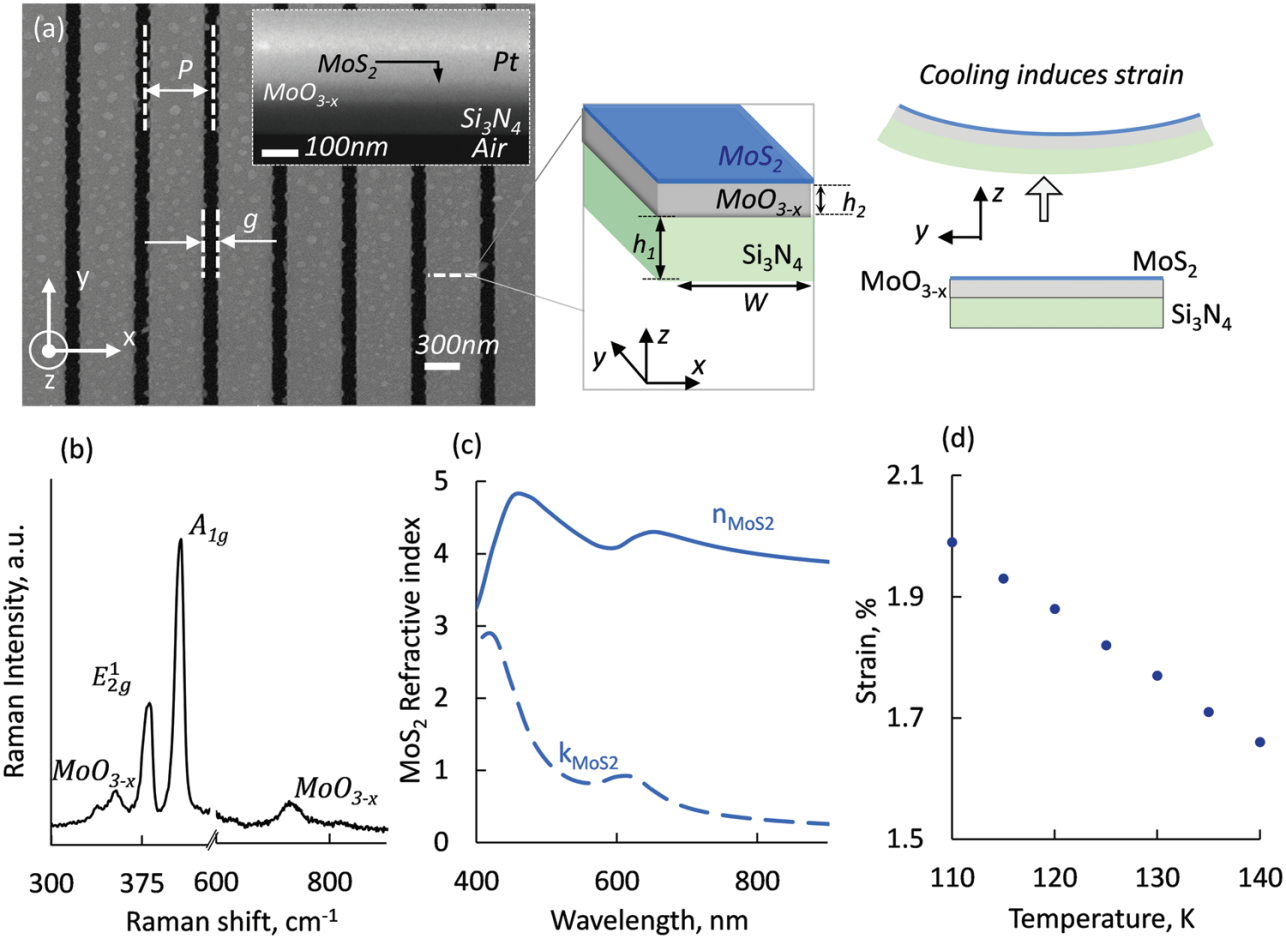
Mechanochromic metasurface. a) Scanning electron microscope image of the metamaterial formed by a nanowire array manufactured on a MoS_2_/MoO_3−_ *_x_*/Si_3_N_4_ trilayer free‐standing membrane, schematic of a single nanowire [*P* = 500, *g* = 100, *h*_1_ = 90, *h*_2_ = 50, *h*_3_ = 5, *W* = 400 nm], scale bar 300 nm. Inset: cross section of the trilayer sample, coated with platinum to improve contrast. b) Raman spectra of metamaterial shows the composition of the sample; c) ellipsometric data of a few layer MoS_2_ film; d) strain induced in a single nanowire upon cooling, deformation of the nanowire is caused by the large thermal expansion mismatch between Si_3_N_4_ and MoO_3−_ *_x_*.

**Figure 2:**
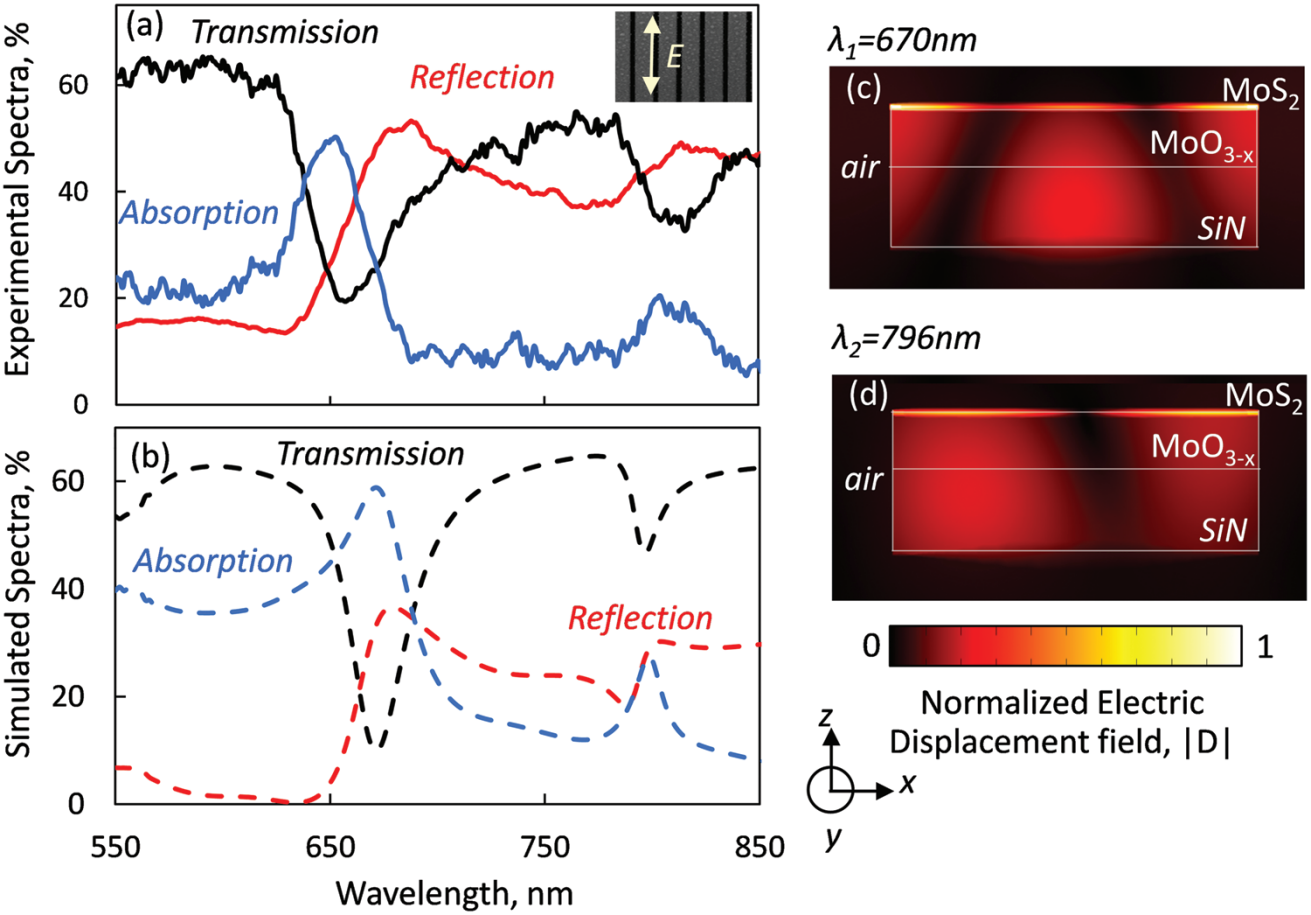
Optical properties of mechanochromic metasurface. a) Reflection, transmission, and absorption spectra of mechanochromic metamaterial under linear polarized illumination, as indicated on inset to plate. b) Numerically simulated reflection, transmission, and absorption spectra of the metamaterial. c,d) Numerically simulated distribution of the electromagnetic field in the metamaterial nanowire. Color maps show the magnitude of the electric displacement field in the *x*–*z* plane.

Measured and computed optical properties of the metamaterial are presented in Figure 2. At optical wavelengths longer than the structure period of 500 nm the nanowire array does not scatter light and acts as an optically homogeneous metamaterial layer, that can be fully characterized in terms of its transmission, reflection and absorption. However, the periodic structuring, results in reflection and transmission resonances at 654 and 810 nm for linearly polarized light parallel to the wires, with a quality factor of *Q* ≈ 25. The experimental spectra are well reproduced in computational modelling, as shown in Figure 2b. The field maps in Figure 2c,d reveal that on resonance, displacement currents running through the nanowires with a higher concentration within the high index MoS_2_ part, interfere constructively with incident light and block transmission over this wavelength, as studied in the past.^6^, ^47^, ^48^ Small discrepancies between measured and computed spectra can be attributed to fabrication tolerances and accuracy of the refractive index values used in the modeling.

Optical properties of such metasurfaces are expected to be strongly dependent on temperature, given the stress‐induced modifications of the band structure of MoS_2_ component. Indeed, heat activated nanoscale reconfiguration of the metamaterial induces nanomechanical deformation of the wires. Since the MoS_2_ is much thinner than Si_3_N_4_ and MoO_3−_ *_x_* layers, the thermal expansion mismatch between the Si_3_N_4_ and MoO_3−_ *_x_* is the main mechanism of the bow‐like deformation of the nanowires upon cooling/heating (the thermal expansion coefficients are 2 × 10^−6^ and 6 × 10^−5^ K^−1^,^49^ respectively). This deformation in its own turn leads to the stress‐induced modification of the optical properties of the MoS_2_ layer. Stress‐induced change of optical properties of the wide band gap materials Si_3_N_4_ (*E*_g_ = 4.5 eV)^50^ and MoO_3−_ *_x_* (*E*_g_ = 3.1 eV)^51^ is insignificant in the part of the spectrum of interest.

Our finite element method (FEM) mechanical stress calculations—based on the linear momentum balance equation and the linear stress–strain relation—are displayed in Figure 1d, employing a single nanowire. Strain ε is the ratio of the deflection of the MoS_2_ layer from the neutral plane, *y* over the radius of curvature, *R*, ε = *y*/*R*. They show that the decrease of the temperature on a nanowire array from 300 to 110 K results in a bow‐like deformation of 50 nm that induces a compressive stress of 2.05% to the MoS_2_ layer. This is a finite element method simulation, as a result stress levels may differ upon grown methods of the films and nanostructuring techniques.

Since thermal cooling of the metasurface induces mechanical strain upon the MoS_2_ layer, the transmission spectra were measured at different temperatures using a microspectrophotometer, to test its mechanochromic response. We first studied the induced changes in optical transmission as a function of applied strain. **Figure** **3** presents the optical transmission of the mechanochromic metasurface for various strain levels, at the cooling rate of 5 K min^−1^. We define the relative transmission change Δ*T*/*T*_0_, where Δ*T* = *T*_ε_ – *T*_0_; *T_ε_* being the absolute transmission at an applied strain ε and *T*_0_ the transmission for zero strain. For small strain levels—below 1%—the transmission change is small, however larger strain upon MoS_2_ induces change in Transmission spectra of the sample. At 2% strain the induced change reaches maximum values of 197% and 80% at 657 and 810 nm, accompanied with the sample's color change, inset Figure 3a. Since, some semiconductors show a variation of their bandgap with temperature, we test if the response in color change is related with the amount of stress applied on the metasurface, or due to temperature. Figure 3b shows the relative transmission difference of the metasurface, for several levels of stress reaching up to 200%, while the trilayer films, where no elastic strain exist, provide transmission changes of the level of 2% between room temperature and 110 K as shown in the inset of Figure 3. Furthermore, no change of the lineshape is recorded, since the relative change is flat. In this work, we did not study the limits of elastic deformation and therefore we did not exceed the 2% applied strain level in pursuit of higher transmission change to avoid irreversible nanomechanical deformations. This performance is already exceptionally high as the metasurface's transmission minimum shifts from λ_ο_ = 657 nm to λ_1_ = 681 nm—providing giant mechanochromic sensitivity, which is defined by the ratio of the wavelength shift over the applied strain Δλ/ε [nm%^−1^] and is found to be as high as 12, one order of magnitude larger than other polymeric mechanochromic systems.^24^ More interestingly, our device outperforms recent reports on polymer based mechanochromic devices.^21^ The mechanochromic metasurface demonstrates transmission difference of 200% under 2% strain at 645 nm wavelength. In contrast, in ref. 21 on Figure 1h it is recorded 77% transmission difference under 50% strain at the wavelength of 600 nm, thus our work outperforms on the transmission contrast achieved. At this and slower cooling rates the change in transmission do not show any hysteretic response.

**Figure 3:**
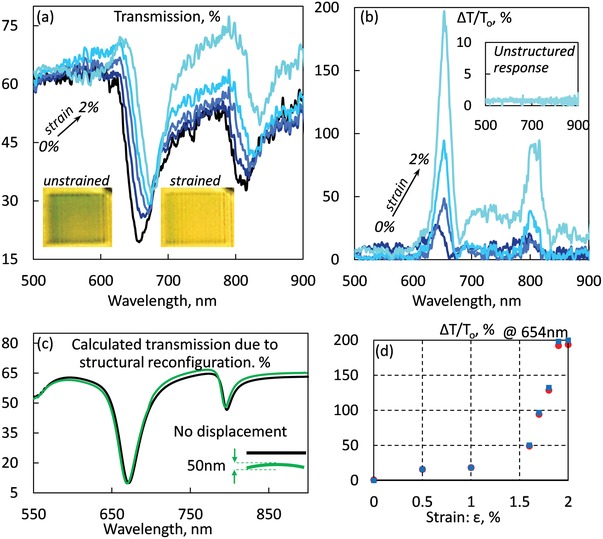
Mechanochromic reconfigurable metasurface. Variation of the optical properties of MoS_2_ metamaterial under stress caused by slow cooling [5 K min^−1^]. a) Spectral dispersion of transmission of the metamaterial for different levels of strain up to maximum strain of 2%. Insets shows the perceived colors of metamaterial sample for zero and 2% strain. b) Relative transmission change for different strain levels. Inset: transmission change of the reference trilayer film. c) Calculated transmission of no displacement, black, and 50 nm displacement of the central part of the sample, green, assuming only structural reconfiguration and no stress dependent refractive index of the nanostructure. d) Reversibility of induced mechanochromic effect—at 654 nm—for the full strain cycle, strain up‐red circular markers, strain down‐cyan rectangular markers.

Upon a faster cooling rate, 15 K min^−1^, a hysteretic behavior of metamaterial's transmission is observed. **Figure** **4**a presents a selection of spectra for different strain levels. For increasing strain, the metasurface demonstrates a red‐shift of its spectrum similar to Figure 3. However, on the level of 1.9% strain the second dip of transmission mode is recorded, as transmission jumps from 31% to 41% for the wavelength of 680 nm, see Figure 4b. In Figure 4b we show the full strain cycle over transmission at the wavelength of 680 nm. Upon decreasing strain—blue line—the optical spectra are different from those of the same strain level for increasing strain—red line. At the level of 1% strain the hysteretic loop closes. Furthermore, we define the differential hysteresis: Δ*h =* Abs[Tr_up_(λ) − Tr_down_(λ)]/Tr_up_ (λ) as the relative transmission difference between the two nonvolatile states for increasing–decreasing strain, with the largest value recorded at the wavelength of 678 nm equal to 47%. This response is mainly driven by the mechanical buckling of the metasurface;^52^, ^53^ an effect accompanying flexible parts when subject to large applied mechanical load and therefore demonstrate mechanical bistability.

**Figure 4:**
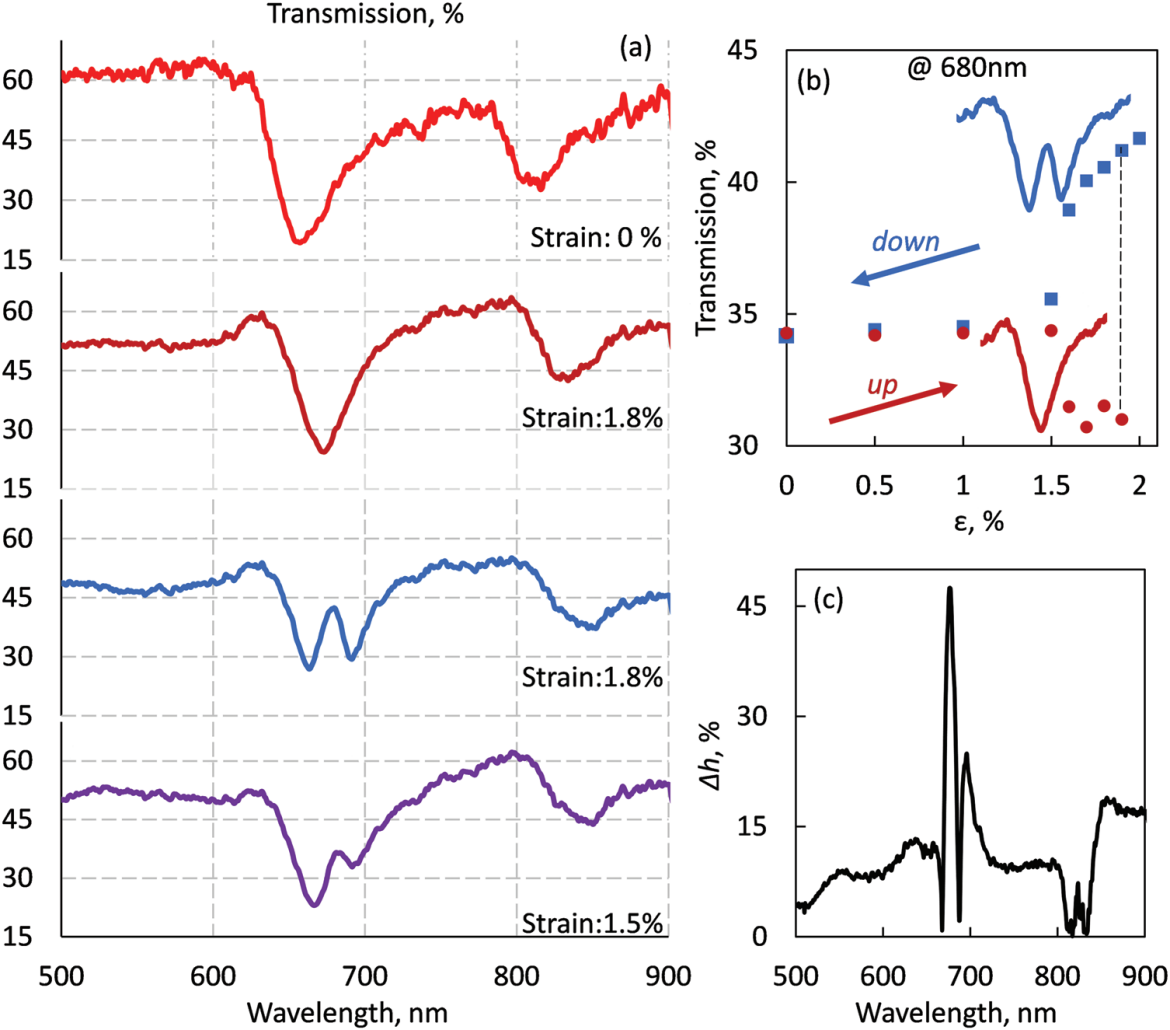
Hysterisis of mechanochromic metasurface. a) Transmission spectra for different strain levels in the regime of rapid cooling [15 K min^−1^]. b) Variation of the metamaterial transmission at 680 nm during the full strain cycle. A hysteresis of optical properties is observed at strain levels exceeding 1.5%. c) Span of the hysteresis loop. Differential hysteresis: Δ*h* = |Tr_up_(λ) − Tr_down_(λ)|/Tr_up_(λ) as a function of wavelength at strain level of 1.8%.

The experimentally observed red‐shift of the optical response of the metamaterial spectrum depending on temperature can be satisfactorily explained in terms of mechanocromism. The red‐shift of the spectrum is related to the mechanochromic nature of device rather than the structural reconfiguration of the sample. We have designed the metamaterial to keep its optical response invariant upon deflection of the nanowires. Upon cooling, nanowires bend in the same fashion, as a result, the metamaterial's response is expected to provide negligible transmission changes for small deformations between neighboring nanowires, since most parameters remain the same such as the distance between the nanowires and the period of the metasurface. This is in contrast to previous works that present substantial changes in the optical properties due to the doubling of metamaterial's period or by controlling the gap between the nanowires.^1^, ^54^, ^55^ Our mechanical FEM calculations (COMSOL) indicate that the midpoint of each nanowire is displaced out‐of‐plane by ≈50 nm when strain is at 2% and temperature at 110 K. For this deflection, the optical FEM modeling indicates that solely the 50 nm mechanical deformation of the central part of the array of the nanowires can induce relative transmission changes smaller than 5% (see Figure 3c). Moreover, the simulation without MoS_2_ film reveals a similar response with Figure 3c, with the transmission difference at 645 nm to be less than 3%, in contrast to experimentally observed change of 200%, at Figure 3b. Furthermore, the fact that the transmission change is nonlinear with the applied strain, Figure 3d as well as the observed hysteresis related to the applied stress rate over an increasing‐decreasing thermal cycle, Figure 4, further verifies the mechanical nature of the effect over any thermal effects, that might exist, negligible in the current study.

In summary, we have reported a new mechanism to tune the optical properties of photonic metamaterials. We have studied the mechanochromic response of MoS_2_ metasurfaces and identified the conditions under which they can operate either as photonic filters or switches. The proposed device provides continuous and reversible optical tuning of visible transmission with relative transmission change of more than 190% actuated by a strain level of 2%, which is translated into great mechanochromic sensitivity. For example, materials with larger thermal expansion coefficient difference or longer nanowires can improve mechanochromic sensitivity. Moreover, new designs of mechanochromic metasurfaces should be developed to improve the bandwidth of the response. Even though we apply a heat‐actuated method to induce stress on the mechanochromic metasurfaces, equivalent strain effects are also achievable via electrically means in nanomechanical metamaterials, such as electrostriction.^56^ We also believe that other types of transition metal dichalcogenides should be studied as strain dependent refractive index materials for metamaterials, targeting different operational wavelengths. Overall, the use of photonic metasurface technology for creating mechanochromic devices based on transition metal dichalcogenides, can be used as a general platform for force responsive materials with optical readouts and form a new family of ultrathin color displays and multifunctional devices.

## Experimental Section

*Metamaterial Fabrication*: Nanograting metamaterial patterns, with a fixed gap width *g* = 100 nm and period *P* = 500 nm, covering an area of ≈22 µm x 25 µm, were directly etched via focused ion beam (FIB) milling, using a FEI Helios NanoLab 600 dual beam system, on a commercially available 90 nm thick Si_3_N_4_ membrane from Norcada Inc. Then, an APCVD synthesis method was used for the deposition of the MoS_2_ on the prepatterned membrane, with two separate precursors: MoO_3_ and sulfur loaded in two 3 cm quartz tubes in a CVD chamber. A silicon nitride membrane was placed at the center of the furnace and a quartz boat containing the MoO_3_ precursor was placed upstream at a distance of 3 cm. The sulfur powder is placed in a separate quartz boat outside the furnace at a distance of 30 cm with controlled ambient temperature. Prior to the deposition, the quartz tube was flushed with 500 sccm of Ar gas for 1 h. The temperature of the furnace was first ramped up to 500 °C with a rate of 20 °C min^−1^ and subsequently up to 700 °C with 4 °C min^−1^ under constant flow of Ar at 200 sccm allowing the evaporation and deposition of MoO_3_. At the temperature of 700 °C the sulfur zone was heated up to 170 °C for 15 min. During the process, a sulfur rich time window, the as‐deposited MoO_3_ was partially converted to MoO_3−_ *_x_* and MoS_2_ progressing in a layer by layer manner while MoS_2_ in the gas phase was codeposited. After the 15 min time window, the gradually sulfur deficient environment resulted in the deposition of a metal oxide rich layer.

*Variable‐Angle Spectroscopic Ellipsometry*: The complex relative permittivity/refractive index APCVD deposited MoS_2_ was evaluated by spectroscopic ellipsometry (J. A. Woollam 4000) over the wavelength range from 400 to 900 nm.

*Numerical Simulations*: Full‐wave electromagnetic simulations of the metamaterial structure, based on the geometry presented in Figure 1a, were performed using the finite element method in COMSOL Multiphysics. Calculations employ periodic boundary conditions in the *x* and *y* directions (i.e., effectively assuming an infinite array of infinitely long nanowires). They utilize refractive indices for silicon nitride and MoO_3−x_ assumed to be nondispersive and equal to 2, while for MoS_2_ ellipsometry data used are presented in Figure 1c. Furthermore, numerical spectra were integrated over the numerical aperture of the objective in the experiment, assuming linearly polarized plane wave illumination; nanowire mechanical deformation was obtained from finite element models of a single, isolated 22 µm long wire with fixed ends and rectangular cross sections as presented in Figure 1. These assumed Young's moduli *E* and density ρ values for Si_3_N_4_, MoO_3−_ *_x_* and MoS_2_: *E*_SiN_ = 200 GPa; *E*_MoO_ = 80 GPa; *E*_MoS_ = 320 GPa; ρ_SiN_ = 3170 kg m^−3^, ρ_MoO_ = 4690 kg m^−3^, ρ_MoS_ = 5060 kg m^−3^.

*Raman Spectroscopy*: Raman measurements under 532 nm laser excitation indicate the deposition on the prepatterned membrane of both MoO_3−_ *_x_* and MoS_2_. The bulk MoS_2_ was confirmed by the Raman mode at ≈381 cm^−1^ and A_1g_ mode at 407.5 cm^−1^;^44^ and the MoO_3−_ *_x_* the Raman peaks at 353 and 733 cm^−1^.^45^

*Microspectrophotometry (Including Low Temperature Measurements)*: Transmission and reflection spectra (Figure 2) were obtained using a microspectrophotometer (CRAIC QDI2010), with a 11 µm × 11 µm sampling aperture via a 15× objective with NA 0.28. All data are normalized to reference levels for air (100% transmission), a silver mirror (high reflector), and a “Vantablack” vertically aligned carbon nanotube array (zero reflection/transmission), and averaged over 15 repeated measurement cycles, each with a 500 ms integration time. For the low temperature measurements, a cryogenic stage (model No THMS600) with temperature control was used. Pressure level is monitored throughout the experiment at 10^−3^ mbar. Measurements performed while temperature level were stabilized with fluctuation of less than 0.1 K.

## Conflict of Interest

The authors declare no conflict of interest.
